# 3D Spheroid Cultures for Salivary Gland Tissue Engineering: Effects of Fibroblast on Epithelial Cell Function

**DOI:** 10.3390/life15040607

**Published:** 2025-04-05

**Authors:** Lan Thi Phuong Nguyen, Joo Hyun Kim, Jiwon Son, Sung Sik Hur, Minyong Lee, Hyung Kwon Byeon, Jin-Young Kim, Myung Jin Ban, Joo Hyun Kim, Man Ryul Lee, Jae Hong Park, Yongsung Hwang

**Affiliations:** 1 Soonchunhyang Institute of Medi-Bio Science (SIMS), Soonchunhyang University, Cheonan 31151, Republic of Korea; nguyenthiphuonglan014@gmail.com (L.T.P.N.); noirsky@naver.com (J.H.K.); pearl3717@naver.com (J.S.); sstahur@gmail.com (S.S.H.); alsdyd0365@naver.com (M.L.); 2 Department of Integrated Biomedical Science, Soonchunhyang University, Asan 31538, Republic of Korea; 3 Department of Otorhinolaryngology-Head and Neck Surgery, College of Medicine, Soonchunhyang University Cheonan Hospital, Cheonan 31151, Republic of Korea; mjbanent@schmc.ac.kr; 4 Department of Otorhinolaryngology-Head and Neck Surgery, College of Medicine, Soonchunhyang University Seoul Hospital, Seoul 04401, Republic of Korea; ewellcastle@schmc.ac.kr; 5 Division of Respiratory Allergy and Critical Care Medicine, Department of Internal Medicine, Soonchunhyang University Cheonan Hospital, Cheonan 31151, Republic of Korea; 109947@schmc.ac.kr; 6 Department of Otorhinolaryngology, Yongin Severance Hospital, Yonsei University College of Medicine, Yongin 16995, Republic of Korea; hilguy@yuhs.ac; 7 Department of Stem Cell and Regenerative Biotechnology, Konkuk Institute of Science and Technology, Konkuk University, Seoul 05029, Republic of Korea

**Keywords:** 3D spheroids, epithelial cells, fibroblasts, salivary glands, senescence

## Abstract

Three-dimensional (3D) spheroid cultures are crucial for modeling salivary gland (SG) morphogenesis and advancing regenerative medicine. This study evaluated the effects of varying ratios of mouse SG-derived epithelial cells co-cultured with human dermal fibroblasts (hDFs), identifying a 2:1 ratio (spheroids containing 67% EpCAM^pos^ cells with 33% hDFs) as optimal for preserving native SG-derived epithelial cell phenotypes. At this ratio, 67% EpCAM^pos^ spheroids maintained structural integrity and demonstrated a significant reduction in apoptosis and senescence markers, specifically, cleaved caspase-3 (Cc3) and Serpine1, alongside an enhanced expression of the progenitor marker Keratin 5 (KRT5). This highlights the pivotal role of fibroblasts in supporting epithelial cell function in 3D cultures. These spheroids provide a useful model for developing SG tissues that closely mimic physiological properties. Despite promising results, these findings are preliminary and require further validation under diverse conditions and across different SG models.

## 1. Introduction

Salivary glands (SGs) are integral to oral health, facilitating taste, chewing, swallowing, and lubrication of the oral mucosa by secreting saliva, which also serves as a barrier against pathogens [1]. Disorders such as SG cancer, irradiation-induced SG dysfunction, and Sjögren’s syndrome drastically reduce SG secretory capacity, significantly impacting patient quality of life [2,3,4]. While sialendoscopy, an extensively used technique for diagnosing and treating salivary gland disorders, offers a valuable interventional approach to alleviate symptoms, it has limitations in fully restoring gland functionality [5]. Therefore, cell-based therapies have emerged as promising regenerative medicine approaches to restore SG functionality in these contexts [6,7]. While primary two-dimensional (2D) cultures have provided valuable insights into SG regeneration, they fail to mimic essential cell–cell and cell–matrix interactions, resulting in altered cell characteristics and the loss of cellular polarity [8,9,10].

To address the limitations inherent in 2D and 3D SG culture, techniques have been developed. These methods more accurately replicate the native tissue architecture and functional dynamics [11]. For instance, Maimet et al. demonstrated that embedding high levels of EpCAM-positive (EpCAM^pos^) cells in Matrigel enhances SG functionality in a murine model of irradiation-induced hyposalivation [12]. Yoon et al. refined this approach by employing neuregulin1 to activate Wnt signaling, combined with A83-01 and Noggin to prevent acinar-to-ductal transitions, and FGF1/FGF7 to maintain the acinar lineage, effectively preserving murine and human SG cell types [13]. However, these models predominantly utilize epithelial cell monocultures and depend on continuous growth factor supplementation to sustain cell functionality, which does not fully replicate the intricate stromal interactions found in native tissues. Notably, during SG morphogenesis, epithelial cells express FGFRb receptors that interact with mesenchymal cell-secreted FGF10, guiding epithelial morphogenesis [14].

Emerging research, such as the work by Kim et al., shows that spheroids incorporating both SG stem cells (SGSCs) and adipose-derived stem cells (ASCs) display significantly enhanced expression of acinar (AQP5) and basal ductal (KRT14) markers compared to spheroids containing only SGSCs [15]. Moreover, human dermal fibroblasts (hDFs), recognized as functional mesenchymal stem cells (MSCs), have been used in 3D cultures. [16]. Franchi-Mendes et al. successfully developed triple heterotypic spheroids consisting of breast cancer epithelial cells (HCC1954), hDF, and endothelial cells, finding an optimal cell ratio that favored endothelial proliferation [17].

Despite these advances, the optimal ratio of fibroblasts to SG epithelial cells required to maintain specific SG tissue characteristics remains underexplored. Thus, in this study, we aim to develop and optimize a co-culture spheroid model using mouse submandibular gland (mSMG)-derived EpCAM^pos^ cells and hDF to preserve salivary gland-specific properties effectively.

## 2. Materials and Methods

### 2.1. Isolation of SG-Derived Epithelial Cells

SG tissues from 14 submandibular glands (SMGs) of 7 male C57BL/6 mice (8–10 weeks old) were harvested and immediately placed in Petri dishes containing 5 mL of DMEM/F-12 (cat#11320-033; Gibco, Thermo Fisher Scientific, Waltham, MA, USA) supplemented with 1% penicillin–streptomycin (cat#1514022; Gibco, Thermo Fisher Scientific, Waltham, MA, USA). Tissues were minced into fragments of approximately 1 mm³ under sterile conditions. The minced tissues were enzymatically digested in HBSS (cat#14025076; Thermo Fisher Scientific, Waltham, MA, USA), calcium- and magnesium-free, with no phenol red, and with 2 mg/mL collagenase type 2 (cat#LS004177; Worthington Biochemical Corporation, Lakewood, NJ, USA), 1 mg/mL hyaluronidase (cat#07461; STEMCELL Technologies, Vancouver, Canada), and 12.5 μM CaCl_2_ at 37 °C in a shaking incubator for 40–60 min.

After digestion, the suspension was centrifuged at 800× *g* for 5 min, and the pellet was resuspended in HBSS and filtered through a 100 μm cell strainer (cat#93100; SPL Life, Pocheon-si, South Korea) to remove undigested tissue and cell clumps. The filtrates were further purified by treatment with Red Blood Cell Lysing Buffer Hybri-Max™ (cat#R7757; Sigma-Aldrich, St. Louis, MO, USA) (1 mL per 10⁶ cells) for 1 min at room temperature and subsequently passed through a 40 μm strainer (cat#93040; SPL Life Sciences, Pocheon, Republic of Korea) to obtain a single-cell suspension. All procedures adhered to ethical standards and protocols approved by the Institutional Animal Care and Use Committee of Soonchunhyang University (protocol SCH17-0003).

### 2.2. Magnetic-Assisted Cell Sorting (MACS) of EpCAM^pos^ Cells

The single-cell suspension was incubated with anti-EpCAM magnetic beads (130-061-101, Miltenyi Biotec, Bergisch Gladbach, Germany) for 20 min at room temperature using a bead-to-cell ratio of 1:5 in MACS buffer (0.5% BSA, 2 mM EDTA in PBS). After incubation, the cells were passed through an MS column to isolate EpCAM^pos^ cells. The positive fraction was collected and cultured in SG medium comprising DMEM/F-12, 1% penicillin–streptomycin, 10% FBS, and a mix of growth factors: 20 ng/mL of EGF (PHG0313, Thermo Fisher Scientific), 20 ng/mL of bFGF (100-18B, PeproTech, Cranbury, NJ, USA), N2 supplement (17,502,048, Thermo Fisher Scientific), 10 μg/mL of insulin (cat# i5500, Sigma-Aldrich, St. Louis, MO, USA), and 1 μM of dexamethasone (cat#D2915, Sigma-Aldrich).

Additionally, 10 μM of Y27632 (cat#129823, Biogems, Westlake Village, CA, USA) and 1 μM of A83-01 (transforming growth factor β inhibitor, cat#2939, Tocris Bioscience, Bristol, UK) were added as required to promote proliferation. A SG medium was previously developed to support the expansion of SG epithelial cells under culture conditions [12,13].

### 2.3. Formation of Multicellular 3D SG Spheroids

EpCAM^pos^ cells were co-cultured with hDFs at proportions of 0%, 25%, 33%, 67%, 75%, and 100% mSMG-derived EpCAM^pos^ cells. Human dermal fibroblasts (hDF) were employed for co-culture experiments due to their compatibility with our analytical techniques; specifically, hDFs do not interfere with the quantitative PCR (qPCR) analysis aimed at detecting mouse-specific epithelial markers. Multicellular spheroids were formed in an ultra-low attachment 96-well plate (cat#7007, Corning, New York City, NY, USA) by centrifuging the cells at 1000 rpm for 5 min at room temperature to facilitate spheroid formation. The spheroids were cultured for up to five days in a SG medium. The seeding density for all experiments was set to 10,000 cells per spheroid (except for qPCR).

### 2.4. Cell Pre-Labeling with Fluorescent Dyes

To visualize the distribution of cells within the 3D spheroids, hDF and mouse SG-derived epithelial cells were pre-labeled with CellTracker Red CMTPX (cat#34552, Invitrogen) and CellTracker Green CMFDA (cat#C7025, Invitrogen, Waltham, MA, USA) for 30 min at 37 °C before spheroid formation.

### 2.5. Cell Viability Assay

The proliferation of SG spheroids was evaluated using a CellVia assay kit (cat# LF-EZ1001, AbFrontier, Republic of Korea). SG spheroids were formed in a 96-well plate and treated with the water-soluble tetrazolium salt WST-1 (4-[3-(4-Iodophenyl)-2-(4-nitro-phenyl)-2H-5-tetrazolio]-1,3-benzene sulfonate) at 10% of the total volume for 2 h at 37 °C. After incubation, the optical density was measured at a wavelength of 450 nm using a microplate reader.

### 2.6. Spheroid Morphology Analysis

Spheroid morphology was monitored and imaged on days 0, 1, 3, and 5 using 10× fluorescence and brightfield microscopy (N.A. = 0.3, pixel size = 0.617 µm/px; model: EVOS M7000; Invitrogen, Carlsbad, CA, USA). Morphological parameters, including volume (VOL) and circularity (CR), were determined using custom image processing programs developed in MATLAB (MathWorks, Natick, MA, USA; version R2023b), following a previously described methodology [18].

Briefly, spheroid boundaries were identified using regions of interest (ROIs) and Otsu’s thresholding algorithm [19]. Volume (VOL), a measure of spheroids size, was inferred from 2D images by assuming a spherical shape—i.e., equal diameters in the x, y, and z directions—as shown below. Spheroid diameters were estimated from the 2D area (AR).

$$VOL=\frac{4}{3\sqrt{\pi }}\;{\left(AR\right)}^{1.5}$$

The circularity ratio (CR), an indicator of spheroid roundness, was defined as the square of the ratio between the perimeter of a perfect circle and the actual perimeter (PR) of the object. CR is a unitless value ranging from 0 to 1, where 1 indicates a perfect circle, and 0 corresponds to an object with an infinite perimeter, signifying extreme irregularity or spikiness.

$$CR=\frac{4\pi \;*Area}{{PR}^{2}}$$

### 2.7. Immunofluorescent Staining

On day 3 after spheroid seeding, the spheroids were collected in 1.5 mL tubes, and excess media were removed. The spheroids were fixed with 4% PFA for 15 min at room temperature and washed three times with PBS (10 min each). The spheroids were then blocked with 1% BSA, 0.05% PBST, and 0.5% Triton X for 2 h at RT. Subsequently, the samples were incubated overnight at 4 °C with the following primary antibodies: anti-keratin 5 (cat#ab52635, Abcam, Cambridge, UK, 1:200), anti-pan cytokeratin (cat#sc-8018, Santa Cruz Biotechnology, Dallas, TX, USA, 1:100), anti-keratin 18 (cat#ab668, Abcam, 1:200), anti-E-cadherin (cat#3195S, Cell Signaling Technology, Danvers, MA, USA, 1:200), and anti-fibronectin (cat#ab2413, Abcam, 1:200).

The next day, the spheroids were washed twice with 0.1% PBST (10 min per wash) and incubated with secondary antibodies—anti-rabbit AlexaFluor 488 (cat#A11008, Invitrogen) and anti-mouse AlexaFluor 555 (cat#A21422, Invitrogen)—for 2 h at room temperature. After washing, the spheroids were treated with the Cytovista tissue clearing kit (cat#V11322, Thermo Fisher Scientific) for 20 min at room temperature to enhance fluorescent signals. Imaging was performed using a confocal microscope (LSM 710; Carl Zeiss, Oberkochen, Germany) at the Soonchunhyang Biomedical Research Core Facility of the Korea Basic Science Institute.

### 2.8. qPCR

Each spheroid contained approximately 20,000 cells, and 15–16 spheroids were pooled per group for qPCR analysis. One milliliter of TRIzol reagent (cat# 15596026, Invitrogen, Carlsbad, CA, USA) was added to each tube. The spheroids were homogenized using a Bead Ruptor Elite Homogenizer (OMNI International, Kennesaw, GA, USA). Total RNA was extracted and reverse-transcribed into complementary DNA (cDNA) using the ReverTra Ace qPCR RT Master Mix with gDNA Remover (cat# KMM-101, TOYOBO, Osaka, Japan) according to the manufacturer’s protocol. qPCR was performed with the SYBR Green Real-Time PCR Master Mix (cat# 4472918, TOYOBO, Osaka, Japan) on a QuantStudio™ 5 Real-Time PCR System (Applied Biosystems, Foster City, CA, USA) at the Soonchunhyang Institute of Medi-Bio Science. Target gene expression levels were normalized to those of glyceraldehyde-3-phosphate dehydrogenase (GAPDH). ΔCt values were calculated as CT_target_ − CT_GAPDH_, and relative fold changes were determined using the 2^−ΔΔCt^ method. The primer sequences used in this study are listed in Table 1.

### 2.9. Statistical Analysis

Bar and line graphs represent the mean and standard error values. A one-way analysis of variance (ANOVA) with Tukey’s multiple comparison test was conducted to evaluate differences among multiple groups. GraphPad Prism software version 8 was used, and *p*-values were reported as follows: * *p* < 0.05; ** *p* < 0.01; and *** *p* < 0.001 for three biological replicates.

## 3. Results

### 3.1. Fibroblasts Enhance Structural Integrity in Short-Term SG-Derived Spheroid Cultures

**Figure 1:**
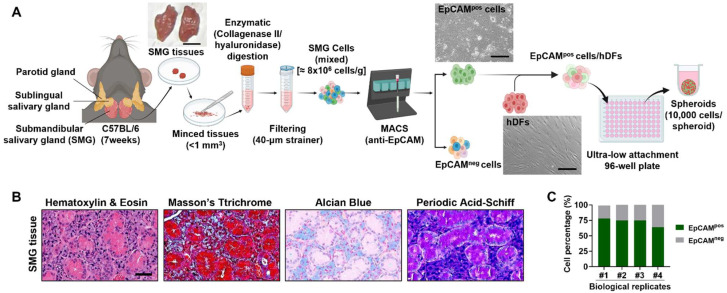
Experimental setup and isolation of EpCAMpos cells from salivary glands. (**A**) Schematic representation of the experimental procedure for isolating cells from SG. The SG tissue is dissociated, and EpCAMpos and EpCAMneg cells are separated using magnetic-activated cell sorting (MACS). EpCAMpos cells were then mixed with human dermal fibroblasts (hDFs) with varying ratios to form spheroids using an ultra-low attachment 96-well plate. (**B**) Histological images of normal SG tissue, showing the typical architecture of the gland and the distribution of cells within the tissue. Scale bar: 100 µm. (**C**) Bar graph showing the percentage of EpCAMpos cells isolated in various independent MACS experiments. The schematic diagram is drawn by BioRender (www.biorender.com).

We developed an advanced 3D culture system by co-culturing SG-derived epithelial cells with fibroblasts, hypothesizing that multicellular spheroids would better preserve tissue characteristics than monocultures of epithelial cells. Initially, we isolated EpCAM^pos^ and EpCAM^neg^ cells, performing histological analyses to examine SG tissue structure (Figure 1A,B). Magnetic-activated cell sorting (MACS) revealed that EpCAM^pos^ cells constituted 64–78% of the total cell population, closely reflecting their proportion in native tissues (Figure 1C).

**Figure 2:**
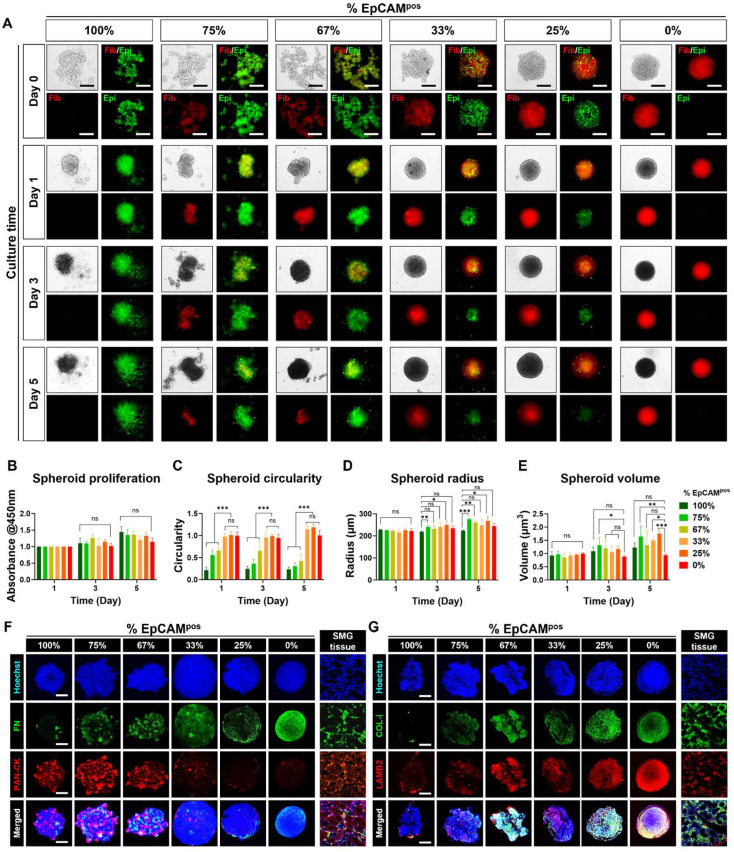
Characterization of epithelial and fibroblast distribution, viability, and morphology in SG spheroids. (**A**) Cell tracker experiments revealing the distribution of the two cell types within SG spheroids during 5 days of culture. Scale bar: 300 μm. (**B**) Comparison of spheroid viability across different cell ratios. Morphological comparison of spheroids across different cell ratios, including (**C**) Circularity, (**D**) Radius, (**E**) Volume. (**F**) Immunostaining of fibronectin (representing fibroblasts) and pan-cytokeratin (representing epithelial cells). (**G**) Immunostaining of Collagen I (COL-1) and Laminin B2 (LAMB2) for spheroids. Scale bar: 100 μm. Data are presented as the mean ± SD. * *p* < 0.05; ** *p* < 0.01; *** *p* < 0.001; “ns” indicates no statistical significance, determined by one-way ANOVA.

Different ratios of epithelial cells to fibroblasts were tested for spheroid formation (Figure 1A). Bright-field imaging over days 0, 1, 3, and 5 showed that spheroids became more compact with increasing proportions of fibroblasts (Figure 2A). Spheroids with higher percentages of epithelial cells (75% and 100%) tended to disintegrate, resulting in irregular shapes (Figure 2A). Spheroid viability assessed via the Cell Counting Kit-8 assay indicated no significant differences across ratios (Figure 2B). Morphological analysis revealed distinct structural differences among the spheroids. Spheroids composed entirely of EpCAM^pos^ epithelial cells demonstrated the lowest circularity, with a value of approximately 0.2 ± 0.07, significantly differing from other groups (*** *p* < 0.001). In contrast, spheroids with 0–33% epithelial cells exhibited near-perfect circularity, with circularity indices of approximately 1 ± 0.08, 0.078, and 0.069, respectively (Figure 2C). The fibroblast-only group displayed the smallest spheroid radius, measuring 218.87 ± 3.53 µm on day 3 and 223.74 ± 3.26 µm on day 5, indicative of excellent spheroid compaction (Figure 2D). Conversely, the spheroids composed solely of epithelial cells had larger radii of 234.69 ± 11.19 µm on day 3 and 244.24 ± 11.9 µm on day 5, which can be attributed to reduced cell–cell adhesion, leading to dispersion from the spheroid core (Figure 2D). This dispersion was further evidenced by the significantly lower volumetric measurements of these spheroids, averaging 0.24 ± 0.05 mm³ (Figure 2E).

More importantly, as shown in Figure 2D, spheroids with 67–75% epithelial cells exhibited the highest levels of cytokeratin expression, which was homogeneously distributed throughout the spheroids. Fibroblasts within these spheroids were evenly distributed, as confirmed by fibronectin staining (Figure 2E). Notably, Collagen I and Laminin B2 were highly expressed in co-cultured spheroids, contrasting with their minimal expression in spheroids composed solely of epithelial cells (Figure 2F,G). This differential expression indicates the crucial role of fibroblasts in secreting extracellular matrix proteins. These findings suggest that an optimal ratio of fibroblasts is critical not only for maintaining the structural integrity and round shape of mSMG-derived spheroids but also for supporting uniform cytokeratin expression. This likely occurs through the stabilization of epithelial cell distribution and their physical interactions with surrounding fibroblasts, facilitating a more organized tissue architecture.

### 3.2. Optimal Progenitor Marker Expression in Spheroids with 67–75% mSMG-Derived Epithelial Cells

**Figure 3:**
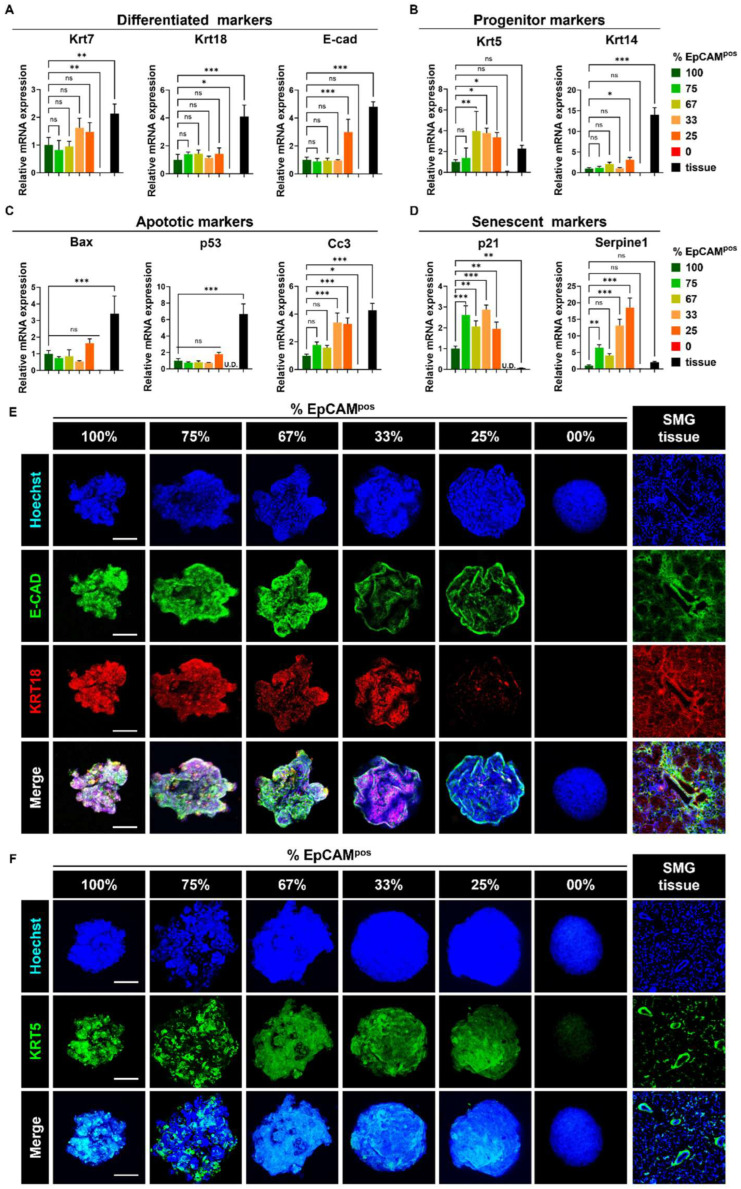
**Spheroids containing 67% EpCAM^pos^ cells exhibit higher expression of progenitor ductal markers and lower p21, Serpine1, and Cc3 expression levels.** (**A**) qPCR analysis of differentiated ductal markers (*Krt7*, *Krt18, E-cad*). (**B**) qPCR analysis of progenitor cell markers (*Krt5, Krt14*). (**C**) qPCR analysis of apoptotic markers (*Bax, P53, Cc3*). (**D**) qPCR analysis of senescence markers (*P21* and *Serpine1).* (**E**,**F**) 3D immunofluorescence (IF) staining to analyze the protein expression levels of E-CAD, KRT18, and KRT5. Scale bar: 100 μm. Data are presented as the mean ± SD. * *p* < 0.05; ** *p* < 0.01; *** *p* < 0.001; “ns” indicates no statistical significance, determined by one-way ANOVA.

In line with our hypothesis that a 67–75% ratio of EpCAM^pos^ cells would optimally preserve progenitor cell markers, closely resembling the cell population in native SG tissue, we assessed the expression levels of both differentiated ductal, epithelial, and progenitor markers. Quantitative analysis revealed no significant differences in the expression of Krt7, Krt18, and E-cad across the experimental groups (Figure 3A). However, spheroids composed of 67% EpCAM^pos^ cells exhibited significantly higher levels of progenitor markers, with Krt5 and Krt14 expressions being three-fold (** *p* < 0.01) and approximately two-fold higher, respectively, compared to those in 100% EpCAM^pos^ spheroids (Figure 3B).

Furthermore, this group displayed notably reduced expression of senescence and apoptotic markers. The expression levels of P21 were relatively lower in the 67% EpCAM^pos^ spheroids compared to the 75% and 33% EpCAM^pos^ groups (*p* = 0.013). Similarly, 67–100% EpCAM^pos^ spheroids exhibited a significantly lower expression level of Cc3 than in the 33% (*p* = 0.01) and 25% EpCAM^pos^ groups (*p* = 0.003). Additionally, Serpine1 expression of 67–100% EpCAM^pos^ spheroids was significantly reduced compared to the 33% (*p* < 0.001) and 25% EpCAM^pos^ groups (*** *p* < 0.001), suggesting a substantial decrease in cellular senescence within spheroids containing 67–75% EpCAM^pos^ cells compared to those with lower proportions (Figure 3C,D).

To corroborate these gene expression findings, we performed immunofluorescence staining for E-CAD, KRT18, and KRT5. The analysis indicated robust expression of E-CAD and KRT18 proteins across the spheroids with 67–100% EpCAM^pos^ cells, whereas no E-CAD expression was detected in fibroblast-only spheroids, and KRT18 was absent in both 25% EpCAM^pos^ and fibroblast-only spheroids (Figure 3E). Importantly, the 67–75% EpCAM^pos^ spheroids demonstrated elevated and well-distributed levels of KRT5 protein compared to spheroids composed solely of EpCAM^pos^ cells (Figure 3F). These results suggest that the inclusion of fibroblasts in specific ratios (67–75% EpCAM^pos^ cells) plays a pivotal role in maintaining progenitor marker expression in SG-derived spheroids.

## 4. Discussion

During natural SG development, epithelial–mesenchymal interactions are pivotal, though their complexity remains not fully understood. Nitta et al. cultured E12 and E13 SMG epithelia without the mesenchyme and investigated the role of EGF and FGF signaling in cleft formation. They discovered that signaling pathways involving epidermal growth factor (EGF) and fibroblast growth factor (FGF) are essential for bud elongation [20]. Similarly, mammary fibroblasts are widely recognized as key regulators of mammary epithelial development during puberty, primarily through the secretion of growth factors such as EGF, IGF1, FGFs, and WNTs [21,22,23,24]. These fibroblasts also secrete ECM proteins, including collagens and fibronectin, regulated by FGF2, which further contribute to the regulation of epithelial cell polarity and morphogenesis [25,26], and broader cellular functions such as proliferation and apoptosis [27].

Given these multifunctional roles, the involvement of fibroblasts in the self-renewal of primary epithelial cells in vitro has gained substantial recognition. For example, our research group developed a co-culture platform for fibroblasts and intestinal epithelial cells, demonstrating that a layer-by-layer co-culture system can efficiently promote the transfer of small molecules through gap junctions, supporting the survival and function of primary intestinal epithelial cells isolated from the small intestine and colon [28]. Further supporting this, Hosseini et al. showed that embryonic mesenchymal cells could increase AQP5 expression in E16 SG epithelial cells by activating FGF2 receptor signaling through direct cell–cell interactions, whereas soluble FGF2 alone was insufficient [29]. Additionally, Sumba et al. demonstrated that FGF2 and FGF9 signaling in mammary fibroblasts promoted fibroblast-driven branching of epithelial cells, with FGF2 playing a crucial role in regulating ECM composition, stiffness, and organization, which, in turn, influences epithelial morphogenesis [25].

In our study, cell sorting experiments led us to speculate that mSMG-derived spheroids containing 67–75% EpCAM^pos^ cells would display the highest expression of SG tissue-specific markers, reflecting the natural EpCAM^pos^ ratio found in native SG tissue. As expected, 67% of the EpCAM^pos^ spheroids expressed higher levels of progenitor markers (*Krt5, Krt14*) compared to other groups (Figure 3B). In our study, enhanced expression of progenitor cell markers in co-cultured epithelial–fibroblast spheroids, as opposed to spheroids composed solely of epithelial cells, can be attributed to a synergistic effect of several factors. The employment of 3T3 fibroblast feeder cells (J2) and the Rho kinase inhibitor (Y-27632) has been shown to effectively suppress differentiation while promoting proliferation and adhesion of human primary epithelial cells via conditional reprogramming [30]. This modulation is characterized by an upregulation in the phosphorylation of Akt (pT308Akt) and extracellular signal-regulated kinases (pERK), along with a concomitant downregulation of the transforming growth factor-beta (TGF-β) signaling pathway. Moreover, Ligaba et al. elucidated that the observed downregulation in human primary keratinocytes might be linked to the enhanced expression of CHAC1, an inhibitor of Notch signaling, and a reduced expression of Hes4, a Notch pathway target gene. Additionally, their findings suggest that the combined influence of fibroblasts and the ROCK inhibitor not only suppresses TGFβ signaling but also supports the maintenance of pluripotency in induced pluripotent stem cells and embryonic stem cells by modulating the FGF/MEK/ERK signaling cascade [31,32]. Interestingly, our findings suggest that a 67% epithelial to 33% fibroblast ratio can mildly suppress the expression of apoptotic (Cc3) and senescence (Serpine1) markers. This suppression may be due in part to the prevention of detachment-induced apoptosis (anoikis), which typically occurs when epithelial cells lose connection with their extracellular microenvironment [33]. The attachment of epithelial cells to ECM components, which are deposited by fibroblasts, likely mitigates this effect [34]. Additionally, another contributing factor could be the inhibition of TGF-β signaling by fibroblast-secreted FGF2, a pathway well known for its significant roles in promoting apoptosis and senescence [35,36,37].

In our study, we utilized human dermal fibroblasts (hDFs) to explore the functions of mouse SG-derived epithelial cells during cell–cell interactions. The strategic choice of hDFs allowed for clear distinction and analysis of murine epithelial responses, which could be uniquely identified by their mRNA expression without cross-species genetic interference. While this approach provides valuable insights into epithelial–stromal signaling dynamics, it also introduces limitations due to potential species-specific differences that may affect cytokine signaling, cell proliferation, and differentiation. To enhance the translational applicability of our findings and better align them with human physiology, future studies should employ fully human SG-derived models.

## 5. Conclusions

Our study highlights the essential role of fibroblasts in maintaining the SG epithelial cell functions within 3D cultures, providing a reliable model for generating SG spheroids that closely mimic the physiological characteristics of native SG tissues. It is important to note that while our results are promising, they are preliminary and were obtained under a specific set of experimental conditions. More comprehensive studies involving the additional use of specific growth factors for the proliferation and differentiation of SG progenitors are needed to fully elucidate the underlying mechanisms of epithelial–stromal interactions and to validate these findings across a broader range of conditions and SG models. Our findings make a contribution to bioengineered salivary gland tissues in regenerative medicine. The combination of epithelial and stromal cells makes our spheroid model useful for studying salivary gland disorders, drug screening, and developing cell-based therapies for patients with salivary hypofunction due to irradiation or autoimmune disease. Future studies should supplement growth factors or extracellular components to create a fully functional SG organoid to enhance its clinical relevance.

## Figures and Tables

**Table 1:** The primer sequences used in this study.

Primer	Sequences (5’–3’)
*Gapdh*_Forward	TTGATGGCAACAATCTCCAC
*Gapdh*_Reverse	CGTCCCGTAGACAAAATGGT
*E-cad*_Forward	CACCTGGAGAGAGGCCATGT
*E-cad*_Reverse	TGGGAAACATGAGCAGCTCT
*Krt5*_Forward	CTGCTGGAGGGCGAGGAATGC
*Krt5*_Reverse	CCACCGAGGCCACCGCCATA
*Krt7*_Forward	CGCCGCTGAGTGTGGACATCG
*Krt7*_Reverse	CTGGCTGCTCTTGGCTGACTTCTG
*Krt14*_Forward	AGCGGCAAGAGTGAGATTTCT
*Krt14*_Reverse	CCTCCAGGTTATTCTCCAGGG
*Krt18*_Forward	AATCAGGGACGCTGAGACCACA
*Krt18*_Reverse	GCTCCATCTGTGCCTTGTATCG
*p21*_Forward	CCTGGTGATGTCCGACCTG
*p21*_Reverse	CCATGAGCGCATCGCAATC
*Bax*_Forward	TGAAGACAGGGGCCTTTTTG
*Bax*_Reverse	AATTCGCCGGAGACACTCG
*Serpine1*_Forward	CCTCTTCCACAAGTCTGATGGC
*Serpine1*_Reverse	GCAGTTCCACAACGTCATACTCG
*p53*_Forward	ACCGCCGACCTATCCTTACC
*p53*_Reverse	TCTTCTGTACGGCGGTCTCTC
*Cc3*_Forward	GGAGTCTGACTGGAAAGCCGAA
*Cc3*_Reverse	CTTCTGGCAAGCCATCTCCTCA

## Data Availability

The original contributions presented in the study are included in the article. Further inquiries can be directed to the corresponding authors.

## Abbreviations

SG, salivary gland; 3D, three-dimensional; 2D, two-dimensional; mSMG, mouse submandibular gland; hDF, human dermal fibroblasts; SGSC, salivary gland stem cells; ASC, adipose-derived stem cells; MSC, mesenchymal stem cells; Cc3, cleaved caspase-3; Krt, keratin; E-cad, E-cadherin; Bax, BCL2-associated X; EpCAM^pos^, EpCAM-positive.
